# Facile One-Step Hydrothermal Synthesis of Na_3_V_2_(PO_4_)_2_F_3_@C/CNTs Tetragonal Micro-Particles as High Performance Cathode Material for Na-Ion Batteries

**DOI:** 10.3389/fchem.2019.00689

**Published:** 2019-10-18

**Authors:** Hao Guo, Yong Hu, Xiaoping Zhang, Rongliang Zhang, Dong Hou, Yulei Sui, Ling Wu

**Affiliations:** ^1^School of Iron and Steel, Soochow University, Suzhou, China; ^2^Zhangjiagang Campus School of Metallurgical and Material Engineering, Jiangsu University of Science and Technology, Zhenjiang, China

**Keywords:** Na-ion batteries, cathode materials, Na_3_V_2_(PO_4_)_2_F_3_, carbon nanotubes, hydrothermal

## Abstract

In this paper, we report a facile one-step hydrothermal method to synthesize tetragonal Na_3_V_2_(PO_4_)_2_F_3_@C particles which are connected by carbon nanotubes (CNTs) networks, using water as hydrothermal solvents. In this strategy, the reduction and crystallization of materials are carried out in the hydrothermal process (180°C, 12 h), no additional heat treatment is required. The well-crystallized Na_3_V_2_(PO_4_)_2_F_3_ tetragonal grains (5–10 μm) are coated with amorphous nano-carbon and connected by highly conductive CNTs. The addition of CNTs can not only improve the conductivity of materials but also effectively inhibit the Na_3_V_2_(PO_4_)_2_F_3_ grains over growth. The Na_3_V_2_(PO_4_)_2_F_3_@C/CNTs composite possesses very flat charge/discharge platforms of 3.6 and 4.1 V. The sample exhibits an initial discharge specific capacity of 120.2 and 74.3 mAh g^−1^ at 0.1 and 10 C rate, respectively, and shows excellent cyclical stability. The composite owns excellent electrochemical performances owing to the three-dimensional highly conductive network which is co-constructed by the CNTs and nano-carbon coating layer.

## Introduction

Recently years, sodium-ion batteries (SIBs) have been receiving great attention due to the low cost and abundant sodium resource. At present, the cathode materials of SIBs mainly include the transition metal oxides (Na_*x*_MeO_2_, Me = Fe, Mn, Co, Ni, and V, etc.) (Delmas et al., 1981; Doeff et al., 1994; Guignard et al., 2013; Vassilaras et al., 2013; Chen et al., 2017, 2018), polyanionic compounds (Na_3_V_2_(PO_4_)_3_, Na_3_V_2_(PO_4_)_2_F_3_, and Na_2_MnPO_4_F, etc.) (Xie et al., 2017; Wu et al., 2018a,b; Zheng et al., 2018; Ge et al., 2019; Leng et al., 2019) and Prussian blue compounds (A_*x*_M_A_[M_B_(CN)_6_]·*y*H_2_O) (Qian et al., 2012; Lee et al., 2013; Wang et al., 2013; Yue et al., 2014). Among these materials, Na_3_V_2_(PO_4_)_2_F_3_ (NVPF) is widely concerned owing to its NASICON structure (Na superionic conductor) and excellent electrochemical performances. Based on the strong induction effect of ${\text{PO}}_{4}^{3-}$ and the strong electronegativity of F^−^, NVPF shows high operating voltages (3.6 and 4.1 V), high energy density (~507 Wh kg^−1^) and excellent thermal stability (Bianchini et al., 2015; Song et al., 2015). Most importantly, the particular NASICON structure can provide three-dimensional (3D) channels for the fast transmission of Na^+^, making NVPF exhibit relatively higher ionic conductivity than many other SIBs cathode materials.

Generally, NVPF materials are mainly synthesized by solid-state reaction method (Gover et al., 2006; Shakoor et al., 2012; Park et al., 2014), spray-drying method (Eshraghi et al., 2017; Shen et al., 2018), sol-gel method (Jiang et al., 2009; Pineda-Aguilar et al., 2017), and hydrothermal method (Serras et al., 2014; Cai et al., 2018; Guo et al., 2018), etc. In order to obtain a well-crystallized NVPF, most of the above mentioned methods usually require high temperature calcination processes (480–850°C). Nevertheless, high temperature will result in that the NVPF particles become irregular, inhomogeneous or even agglomerate together to form larger clusters, which is unfavorable for the Na-ions transmission. Also, the F element may be lost during the calcination process, introducing some impurity phases such as Na_3_V_2_(PO_4_)_3_ (Eshraghi et al., 2017). Furthermore, the excess calcination process will greatly increase the energy consumption. Although several solvothermal routes (Xu et al., 2014; Qi et al., 2015; Zhao et al., 2015; Zhu et al., 2017) have been reported to synthesize NVPF without heat treatment, the required organic solvents [ethanol, acetone, DMF, and acid-base coupling extractant (PN) etc.] have high vapor pressures which are very dangerous. Thus, the vessel used for the solvothermal process must withstand great high pressures, leading to an extra production cost. And the organic raw materials [such as Vanadium (III) acetylacetonate] used in the solvothermal process are also very expensive. In addition, the organic solvents are usually toxic and harmful to the environment. Thus, the solvothermal method is difficult for the practical application.

Therefore, a green and cheap synthetic route for NVPF without additional calcination process should be developed urgently. Here, we propose a water based hydrothermal route to prepare well-crystallized NVPF, without any extra heat treatment process. In this route, all the raw materials (H_3_PO_4_, NH_4_VO_3_, NaF, and oxalic acid) are common and cheap, and NVPF@C tetragons can be one-step synthesized at 180°C with deionized water as solvent. Particularly, here oxalic acid is used as a chelating agent, reducing agent, and carbon source at the same time. In addition, we further add CNTs in the process to obtain NVPF@C/CNTs composite material. The addition of CNTs can not only enhance the conductivity of materials but also restrict the grains over growth. Most importantly, the embedded and wrapped CNTs combined with a uniform nano-carbon layer for NVPF construct a highly conductive 3D network for the transmission of electron and Na^+^. As a result, the micron scale (5–10 μm) NVPF@C/CNTs composite synthesized in this study shows excellent rate capability and cycling performance, which can compete with the nanoscale NVPF materials reported in many other reports (Li et al., 2018a,b). It is well-known that the micron-sized cathode materials are preferred rather than nanosized ones for the commercial batteries (such as LiCoO_2_ and LiNi_*x*_Co_*y*_Mn_*z*_O_2_), as the bigger particles need less binder, solvent and conductive additive to prepare an efficient electrode (Choi and Aurbach, 2016). The micron-sized particles also show higher tap density than the nanosized ones. In a word, this is a green, low cost and efficient water based hydrothermal method for the preparation of NVPF cathode material, and the micron sized NVPF@C/CNTs composite produced by this method is a promising cathode material for SIBs.

## Experimental

### Materials Synthesis

**Figure 1:**
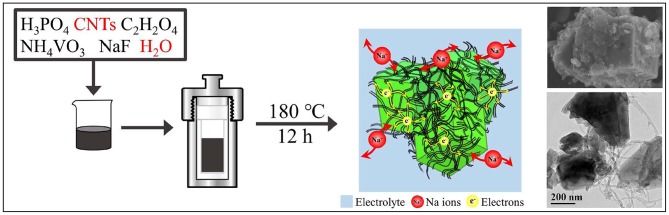
Schematic diagram of synthetic NVPF@C/CNTs cathode material.

The synthesis route of Na_3_V_2_(PO_4_)_2_F_3_@C/CNTs cathode material is shown in Figure 1. Firstly, 0.01 mol H_3_PO_4_ (A.R., Aladdin) and 0.05 g CNTs (commercially available carbon nanotubes) were added into 30 ml deionized water, followed by sonication for 15 min. Then, 0.015 mol C_2_H_2_O_4_ (A.R., Aladdin), 0.01 mol NH_4_VO_3_ (A.R., Aladdin) and 0.015 mol NaF (A.R., Aladdin) were sequentially added, and continuously stirred for 30 min. Thereafter, the mixture was transferred to a Teflon cup of 50 mL inner volume, placed in a stainless steel autoclave and kept in an oven at 180°C for 12 h. After cooling to room temperature, the sample in the kettle was taken out, centrifuged and dried to get Na_3_V_2_(PO_4_)_2_F_3_@C/CNTs composite material (denoted as NVPF@C/CNTs). The Na_3_V_2_(PO_4_)_2_F_3_@C (denoted as NVPF@C) cathode material was prepared without the addition of CNTs by using the same synthesis route.

### Characterization

XRD was carried out on a Rigaku Ultima IV diffractometer using Cu Kα radiation (40 kV); step scan 0.02, 2θ range 10–90°, step time of 2° min^−1^. IR spectrum was tested on a VERTEX70 spectrometer with a transmission mode in the range of 600–2,000 cm^−1^. XPS was performed using a Kratos X-ray photoelectron spectrometer (Axis Ultra-DLD) with Mg Kα radiation (*h*ν = 1283.3 eV), and all the spectra were calibrated with the C 1s peak at 284.8 eV. The carbon content was measured by C–S analysis (Eltar, Germany). SEM (Hitachi SU-5000) was performed to study the morphology of materials. HRTEM and EDX analyses were carried out with a FEI Tecnai G2 F20 microscope at 200 kV.

### Battery Fabrication and Electrochemical Tests

The positive electrode was prepared with the Na_3_V_2_(PO_4_)_2_F_3_ samples, acetylene black and PVDF in the weight ratio of 8:1:1. The N-methylpyrrolidone and Al-foil were used as solvent and current collector, respectively. The metallic sodium-foil and glass fiber membrane (Whatman GF/A) were used as the negative electrode and separator, respectively. The NaClO_4_ (1 M) solution in propylene carbonate (PC) and fluoroethylene carbonate (FEC) (95% PC and 5% FEC in volume) was used as the electrolyte. The CR2025 cells were assembled in an Ar-filled dry glove box (H_2_O and O_2_ < 0.1 ppm). The cells were tested at different C-rates (0.1–10 C, 1C = 120 mA g^−1^) between 2.5 and 4.3 V at ambient temperature. The cyclic voltammetry (CV) was carried out with a CHI-660D electrochemical workstation; potential range 2.5–4.3 V (vs. Na^+^/Na), scan rate 0.1 mV s^−1^.

## Results and Discussion

**Figure 2:**
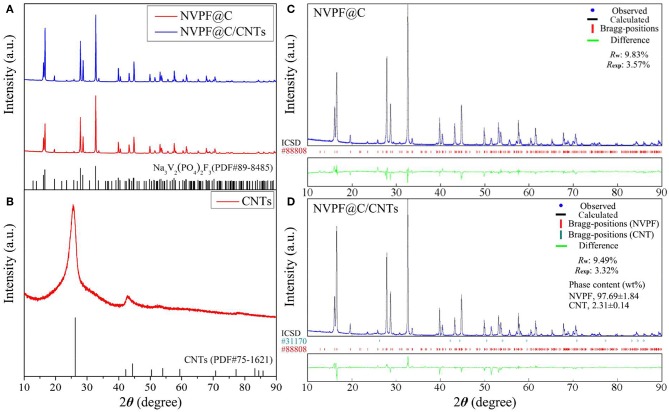
XRD patterns of NVPF@C, NVPF@C/CNTs **(A)** and CNTs **(B)**. Rietveld refinement XRD patterns of NVPF@C **(C)** and NVPF@C/CNTs **(D)**.

Figure 2A shows the XRD patterns of NVPF@C and NVPF@C/CNTs samples. From Figure 2A, it is found that there is no obvious difference between the NVPF@C and NVPF@C/CNTs samples, and all diffraction peaks can be fully indexed as the tetragonal structure of Na_3_V_2_(PO_4_)_2_F_3_ (PDF#89-8485) with space group P4_2_/*mnm*, and no other impurity phases are detected. In addition, the element contents of both samples are also measured. It is found that the molar ratios of Na: V: P: F of NVPF@C and NVPF@C/CNTs are 2.99: 2: 2.01: 3.01 and 3.01: 2: 1.99: 2.98, respectively, which are close to their theoretical values. The above results indicate that the well-crystallized pure phase Na_3_V_2_(PO_4_)_2_F_3_ can be successfully synthesized through water-based hydrothermal method, and the addition of CNTs does not affect the crystal structure of Na_3_V_2_(PO_4_)_2_F_3_. For the sake of contrast, the XRD pattern of CNTs is also shown in Figure 2B. However, those diffraction peaks corresponding to CNTs are not clearly observed in NVPF@C/CNTs. Therefore, in order to further clarify the structure of the samples, the XRD data of NVPF@C and NVPF@C/CNTs samples are refined by Rietveld method, and the refinement results are shown in Figures 2C,D, respectively. As shown, each fitting curve is well-matched with the measured data, and the refinement errors *R*_w_ and *R*_exp_ are acceptable, implying the results are reliable. The refinement results in Figure 2D demonstrate the presence of CNTs and indicate that the phase content of CNTs is about 2.31 ± 0.14 wt%. To verify the accuracy of CNTs content, the samples were subjected to C-S analysis. C-S test results reveal that the carbon content of NVPF@C and NVPF@C/CNTs is 3.04 wt% and 5.21 wt%, respectively. Compared with NVPF@C, the increased carbon content (2.17 wt%) of NVPF@C/CNTs should be ascribed to CNTs. This value (2.17 wt%) is close to the XRD refinement result. The above results reveal that CNTs are successfully compounded with NVPF@C. Table 1 shows the lattice parameters and crystallite size of the two samples. As shown, the lattice parameters of both samples are similar, and all the values are consistent with those reported in the previous references (Gover et al., 2006; Shakoor et al., 2012; Cai et al., 2018). However, the crystallite size (*D* value) of NVPF@C/CNTs is smaller than that of NVPF@C. The results demonstrate that although the addition of CNTs does not affect the crystal structure of NVPF, it can restrict the growth of crystal in some degree, which is of great benefit to obtaining smaller NVPF crystals.

**Table 1:** Lattice parameters and crystallite size of NVPF@C and NVPF@C/CNTs samples obtained from XRD Rietveld refinement.

**Samples**	**Lattice parameters**				**Crystallite size**	**Refinement error**	
	***a*/Å**	***b*/Å**	***c*/Å**	***V*/Å^**3**^**	***D*/nm**	***R*_**w**_/%**	***R*_**exp**_/%**
NVPF@C	9.039	9.039	10.681	872.67	404.79	9.83	3.75
NVPF@C/CNTs	9.038	9.038	10.679	872.31	382.53	9.49	3.32

**Figure 3:**
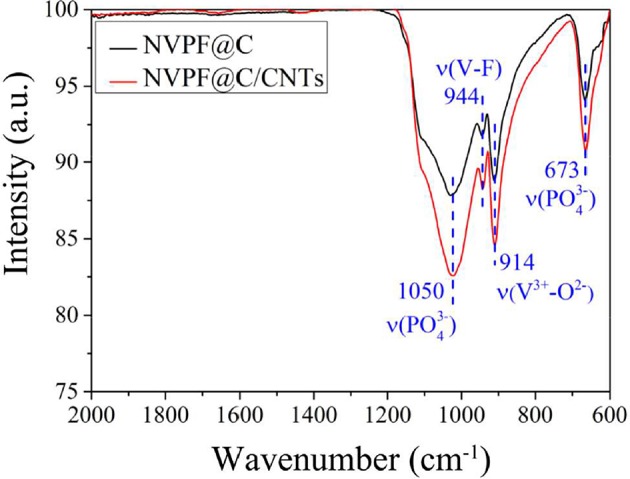
FT-IR spectra of NVPF@C and NVPF@C/CNTs.

Infrared absorption spectroscopy (FTIR) analysis of the NVPF@C and NVPF@C/CNTs samples are shown in Figure 3. It is found that the peaks of both samples appear in the same band, but the absorption peaks of NVPF@C/CNTs are stronger. The band corresponding to the symmetric stretching vibration of ${\text{PO}}_{4}^{3-}$ anion is located at 673 cm^−1^, and the broadband at 1,050 cm^−1^ is ascribed to the asymmetric stretching of ${\text{PO}}_{4}^{3-}$ anion. In addition, the band at 944 cm^−1^ manifests the presence of V–F (Qi et al., 2015; Li et al., 2018b). Moreover, the vibration from V^3+^—O^2−^ bonds in isolated VO_4_F_2_ octahedra, is detected at 914 cm^−1^. While, the typical bands of V^5+^ in the VO_4_F_2_ octahedron are not detected at 760 cm^−1^ and 950 cm^−1^ (Song et al., 2014; Pineda-Aguilar et al., 2017; Cai et al., 2018), meaning that the V^5+^ ions in NH_4_VO_3_ have been reduced to V^3+^.

**Figure 4:**
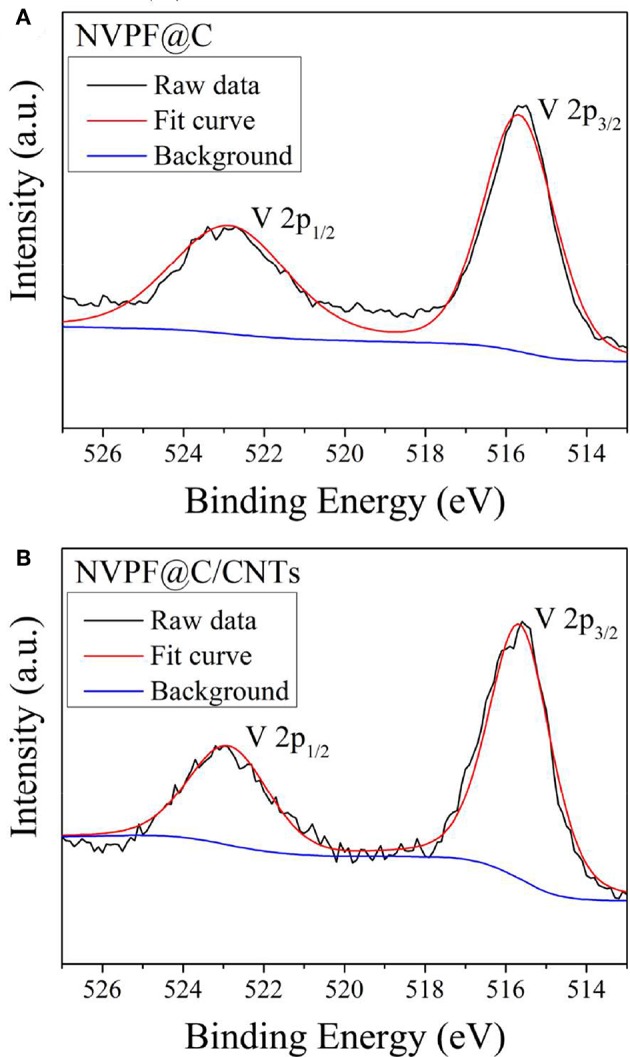
XPS spectra of V 2p for NVPF@C **(A)** and NVPF@C/CNTs **(B)**.

In order to further determine the valence state of V^n+^ in the samples, XPS spectra of V 2p are shown in Figure 4. It can be observed that the peaks corresponding to V 2p_3/2_ and V 2p_1/2_ are present at binding energies of 515.7 and 522.9 eV, which are similar to the binding energies of V^3+^ in the previous literature (Ni et al., 2017; Li et al., 2018a). Based on the above analysis, V^5+^ is successfully reduced to V^3+^ by oxalate acid during the hydrothermal process, and well-crystallized NASICON-type Na_3_V_2_(PO_4_)_2_F_3_ can be obtained by the one-step hydrothermal method. Most significantly, no additional heating process is required.

**Figure 5:**
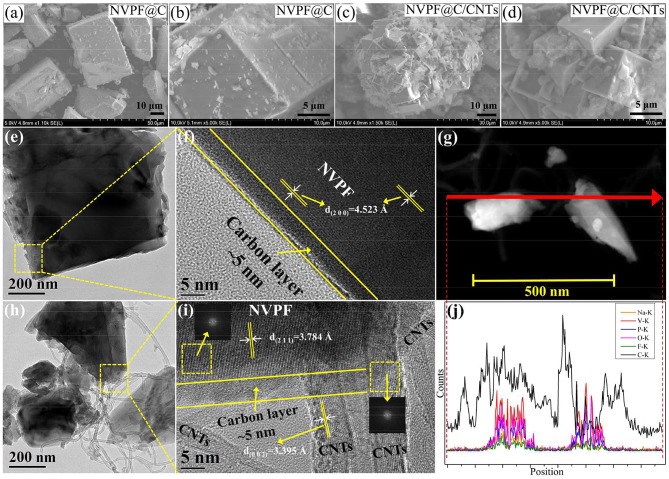
SEM images of NVPF@C **(a,b)** and NVPF@C/CNTs **(c,d)**. TEM and HRTEM images of NVPF@C **(e,f)** and NVPF@C/CNTs **(h,i)**. TEM image of NVPF@C/CNTs **(g)** and corresponding EDS line-scanning curves of Na, V, P, O, F, and C elements **(j)**.

Figures 5a–d shows the SEM images of NVPF@C and NVPF@C/CNTs. As shown, both samples exhibit the tetragonal architecture, but the sample NVPF@C/CNTs shows more regular tetragonal architecture and smaller particle size than NVPF@C. The particle size of NVPF@C and NVPF@C/CNTs is about in the ranges of 30–50 and 5–10 μm, respectively. It is obvious that the NVPF@C grains are too large for the fast insertion/extraction of Na-ions. While the grain size of NVPF@C/CNTs is suitable and very close to those of commercial Li-ion battery materials, such as LiCoO_2_ and LiNi_0.33_Co_0.33_Mn_0.33_O_2_. These changes in the shape and size of NVPF particles are mainly ascribed to the addition of CNTs, which is not only beneficial to restrict the NVPF particles over growth, but also can weave a 3D conductive network between the micro-sized tetragonal grains. This 3D network structure can be expected to greatly improve the conductivity of NVPF cathode materials. The tap densities of NVPF@C and NVPF@C/CNTs are 1.12 and 1.03 g cm^−3^, respectively, which are higher than those of nanoscale powders.

TEM images of the NVPF@C and NVPF@C/CNTs samples are shown in Figures 5e,h, respectively. As can be seen, both samples show tetragon shape, but NVPF@C/CNTs sample exhibits smaller grain size than NVPF@C. Furthermore, NVPF crystals in NVPF@C/CNTs sample are intertwined with CNTs. From the HRTEM images (Figures 5f,i), it can be clearly seen that the surface of the NVPF crystal for both samples are uniformly coated with an amorphous nano-carbon layer (about 5 nm thick). The carbon layer is mainly derived from the carbonization of oxalic acid. The regular lattice fringes corresponding to the (2 0 0) and (2 1 1) crystal planes of NVPF can be observed in NVPF@C and NVPF@C/CNTs samples, respectively. In addition, the lattice fringes of CNTs [*d*_(002)_ = 3.395 Å] can be clearly found in NVPF@C/CNTs, and some lattice fringes of CNTs embed in the lattice fringes of NVPF crystals. The above results confirm that some CNTs are embed into the NVPF crystals and the others wrap around the NVPF@C particles. As a result, the embedded and wrapped CNTs combined with the uniform nano-carbon layer construct a 3D conductive network for the transmission of electron and Na^+^. Moreover, the illustrations in the upper left and lower right areas in Figure 5i are the fast Fourier transform (FFT) of the two selections. The upper left area corresponds to the diffraction spots of the NVPF, and the lower right area corresponds to the diffraction spots of the CNTs. The FFT images further prove the coexistence of NVPF and CNTs. For further study, we perform a EDS linear scanning analysis for NVPF@C/CNTs sample along the line marked in Figure 5g, and the results are shown in Figure 5j. As shown, the Na, V, P, O, and F elements are mainly distributed in the NVPF particles, and the carbon signal in the particles confirms the presence of the carbon layer on the surface of NVPF particles. Significantly, the location of the CNTs shows a very high carbon signal.

**Figure 6:**
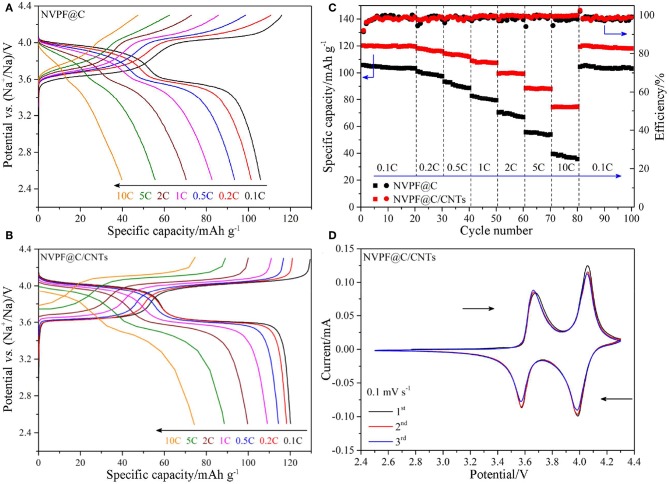
The first charge/discharge curves of NVPF@C **(A)** and NVPF@C/CNTs **(B)** at various C-rates. Cycling performance of NVPF@C and NVPF@C/CNTs at various C-rates **(C)**. CV curves of NVPF@C/CNTs at a scan rate of 0.1 mV s^−1^ **(D)**.

Figures 6A,B shows the charge and discharge curves of the samples at different C-rates. It is obvious that the NVPF@C/CNTs sample shows more flat voltage plateaus, smaller polarization and higher average working voltage than NVPF@C at various C-rates during charge and discharge processes. Furthermore, the initial discharge specific capacity of NVPF@C/CNTs (120.2 mAh g^−1^) is higher than that of NVPF@C (105.8 mAh g^−1^) at 0.1C rate. With the increasing of C-rate (from 1C to 10C), the discharge capacities of NVPF@C/CNTs also exhibit less decay than NVPF@C. For example, the ratio of 10C/0.1C discharge capacity for NVPF@C sample is only 37.6%, while the ratio for NVPF@C/CNTs is as high as 61.8%. Figure 6C shows the cycle performance of the two samples at various C-rates. It is distinct that NVPF@C/CNTs shows better rate capability and cycling performance than NVPF@C. Especially, the NVPF@C/CNTs sample exhibits a capacity retention rate of 99.5% after 20 cycles at 0.1 C rate, and shows almost no capacity fading when cycling at 1C, 2C, 5C, and 10C, respectively. After cycling at various C-rates for 80 cycles, the discharge specific capacity of NVPF@C/CNTs at 0.1C (the 81st cycle) can still holds 99.4% of its initial value of the first cycle (120.2 mAh g^−1^), which indicates that the structure of NVPF is very stable. Moreover, the coulomb efficiency of each cycle is almost 100% at various C rates. Figure 6D presents the CV profiles of NVPF@C/CNTs sample at a sweep rate of 0.1 mV s^−1^. As shown, the sample exhibits the oxidation and reduction peaks of 3.66/3.57 and 4.05/3.98 V, respectively. It should be noted that the 1st, 2nd, and 3rd CV curves overlap closely, which further proves the stable structure of NVPF. The excellent electrochemical performances of NVPF@C/CNTs should be owing to: (1) the well-crystallized NVPF can offer a stable crystal structure; (2) the addition of CNTs can effectively restrict the NVPF grains over growth, which is conducive to the migrating of Na-ions during the charge/discharge processes; (3) the uniform nano-carbon layer, embedded and wrapped CNTs together construct a highly conductive 3D network for the electron transmission.

## Conclusions

Well-crystallized NVPF@C/CNTs cathode material with tetragonal architecture is successfully synthesized by a one-step water based hydrothermal method. In this method, the reduction and crystallization of materials are simultaneously accomplished in the hydrothermal process, no additional heat treatment is needed. The obtained NVPF crystals are coated with a uniform nano-carbon layer, and CNTs are embed into and wrapped around the NVPF grains. Both the coating layer and CNTs weave a highly conductive 3D network for the migration of electron and Na-ion, which leads to the excellent rate capability and cycling performance of the micron-sized (5–10 μm) NVPF@C/CNTs composite. Significantly, the raw materials used in this method are very ordinary, cheap and eco-friendly, the solvent is water and the synthesis temperature is only 180°C. Therefore, we believe that this is a green, low cost, efficient, and facile method for the synthesis of NVPF-based cathode materials.

## Data Availability Statement

All datasets generated for this study are included in the article/supplementary files.

## Author Contributions

HG and YH did the main experiment and wrote the manuscript. XZ, RZ, and YS envolved the discussion of the experiment and revised the manuscript. LW and DH made the research plan. LW also provided the financial support.

### Conflict of Interest

The authors declare that the research was conducted in the absence of any commercial or financial relationships that could be construed as a potential conflict of interest.

## References

[B1] Bianchini M. Fauth F. Brisset N. Weill F. Suard E. Masquelier C. (2015). Comprehensive investigation of the Na_3_V_2_(PO_4_)_2_F_3_-NaV_2_(PO_4_)_2_F_3_ system by operando high resolution synchrotron X-ray diffraction. Chem. Mater. 27, 3009–3020. 10.1021/acs.chemmater.5b00361

[B2] Cai Y. Cao X. Luo Z. Fang G. Liu F. Zhou J.. (2018). Caging Na_3_V_2_(PO_4_)_2_F_3_ microcubes in cross-linked graphene enabling ultrafast sodium storage and long-term cycling. Adv. Sci. 5:1800680. 10.1002/advs.201800680 30250805 PMC6145241

[B3] Chen J. Li L. Wu L. Yao Q. Yang H. Liu Z. (2018). Enhanced cycle stability of Na_0.9_Ni_0.45_Mn_0.55_O_2_ through tailoring O3/P2 hybrid structures for sodium-ion batteries. J. Power Sources 406, 110–117. 10.1016/j.jpowsour.2018.10.058

[B4] Chen J. Zhong S. Zhang X. Liu J. Shi S. Hu Y. (2017). High performance of hexagonal plates P2-Na_2/3_Fe_1/2_Mn_1/2_O_2_ cathode material synthesized by an improved solid-state method. Mater. Lett. 202, 21–24. 10.1016/j.matlet.2017.05.084

[B5] Choi J. W. Aurbach D. (2016). Promise and reality of post-lithium-ion batteries with high energy densities. Nat. Rev. Mater. 1:16013 10.1038/natrevmats.2016.13

[B6] Delmas C. Braconnier J. J. Fouassier C. Hagenmuller P. (1981). Electrochemical intercalation of sodium in Na_*x*_CoO_2_ bronzes. Solid State Ionics 3, 165–169. 10.1016/0167-2738(81)90076-X

[B7] Doeff M. M. Peng M. Y. Ma Y. De Jonghe L. C. (1994). Orthorhombic Na_*x*_MnO_2_ as a cathode material for secondary sodium and lithium polymer batteries. J. Electrochem. Soc. 141, L145–L147. 10.1149/1.2059323

[B8] Eshraghi N. Caes S. Mahmoud A. Cloots R. Vertruyen B. Boschini F. (2017). Sodium vanadium (III) fluorophosphate/carbon nanotubes composite (NVPF/CNT) prepared by spray-drying: good electrochemical performance thanks to well-dispersed CNT network within NVPF particles. Electrochim. Acta 228, 319–324. 10.1016/j.electacta.2017.01.026

[B9] Ge X. Li X. Wang Z. Guo H. Yan G. Wu X. (2019). Facile synthesis of NaVPO_4_F/C cathode with enhanced interfacial conductivity towards long-cycle and high-rate sodium-ion batteries. Chem. Eng. J. 357, 458–462. 10.1016/j.cej.2018.09.099

[B10] Gover R. K. B. Bryan A. Burns P. Barker J. (2006). The electrochemical insertion properties of sodium vanadium fluorophosphates. Na_3_V_2_(PO_4_)_2_F_3_. Solid State Ionics 177, 1495–1500. 10.1016/j.ssi.2006.07.028

[B11] Guignard M. Didier C. Darriet J. Bordet P. Elkaïm E. Delmas C. (2013). P2-Na_*x*_VO_2_ system as electrodes for batteries and electron-correlated materials. Nat. Mater. 12, 74–80. 10.1038/nmat3478 23142842

[B12] Guo B. Diao W. Yuan T. Liu Y. Yuan Q. Li G. (2018). Enhanced electrochemical performance of Na_3_V_2_(PO_4_)_2_F_3_ for Na-ion batteries with nanostructure and carbon coating. J. Mater. Sci. Mater. Electron. 29, 16325–16329. 10.1007/s10854-018-9722-8

[B13] Jiang T. Chen G. Li A. Wang C. Wei Y. (2009). Sol-gel preparation and electrochemical properties of Na_3_V_2_(PO_4_)_2_F_3_/C composite cathode material for lithium ion batteries. J. Alloy. Compd. 478, 604–607. 10.1016/j.jallcom.2008.11.147

[B14] Lee E. Kim D. H. Hwang J. Kang J. S. Minh N. V. Yang I. S. (2013). Soft x-ray absorption spectroscopy study of prussian blue analogue ACo[Fe(CN)_6_]H_2_O nano-particles (A = Na, K). J. Korean Phys. Soc. 62, 1910–1913. 10.3938/jkps.62.1910

[B15] Leng J. Wang Z. Wang J. Wu H.-H. Yan G. Li X.. (2019). Advances in nanostructures fabricated via spray pyrolysis and their applications in energy storage and conversion. Chem. Soc. Rev. 48, 3015–3072. 10.1039/C8CS00904J 31098599

[B16] Li L. Xu Y. Sun X. Chang R. Zhang Y. Zhang X. (2018b). Fluorophosphates from solid-state synthesis and electrochemical ion exchange: NaVPO_4_F or Na_3_V_2_(PO_4_)_2_F_3_. Adv. Energy Mater. 8:1801064 10.1002/aenm.201801064

[B17] Li L. Xu Y. Sun X. He S. Li L. (2018a). High capacity-favorable tap density cathode material based on three-dimensional carbonous framework supported Na_3_V_2_(PO_4_)_2_F_3_ nanoparticles. Chem. Eng. J. 331, 712–719. 10.1016/j.cej.2017.09.012

[B18] Ni Q. Bai Y. Wu F. Wu C. (2017). Polyanion-type electrode materials for sodium-ion batteries. Adv. Sci. 4:1600275. 10.1002/advs.201600275 28331782 PMC5357992

[B19] Park Y. U. Seo D. H. Kim H. Kim J. Lee S. Kim B. (2014). A family of high-performance cathode materials for Na-ion batteries, Na_3_(VO_1−x_PO_4_)_2_F_1+2x_ (0 ≤ *x* ≤ 1): combined first-principles and experimental study. Adv. Funct. Mater. 24, 4603–4614. 10.1002/adfm.201400561

[B20] Pineda-Aguilar N. Gallegos-Sánchez V. J. Sánchez E. M. Torres-González L. C. Garza-Tovar L. L. (2017). Aluminum doped Na_3_V_2_(PO_4_)_2_F_3_ via sol–gel Pechini method as a cathode material for lithium ion batteries. J. Sol Gel Sci. Technol. 83, 405–412. 10.1007/s10971-017-4398-8

[B21] Qi Y. Mu L. Zhao J. Hu Y. S. Liu H. Dai S. (2015). Superior Na-storage performance of low-temperature-synthesized Na_3_(VO_1−−x_PO_4_)_2_F_1+2x_ (0 ≤ *x* ≤ 1) nanoparticles for Na-ion batteries. Angew. Chem. Int. Ed. 54, 9911–9916. 10.1002/anie.201503188 26179243

[B22] Qian J. Zhou M. Cao Y. Ai X. Yang H. (2012). Nanosized Na_4_Fe(CN)_6_/C composite as a low-cost and high-rate cathode material for sodium-ion batteries. Adv. Energy Mater. 2, 410–414. 10.1002/aenm.201100655

[B23] Serras P. Palomares V. Rojo T. Brandc H. E. A. Sharma N. (2014). Structural evolution of high energy density V^3+^/V^4+^ mixed valent Na_3_V_2_O_2x_(PO_4_)_2_F_3–2*x*_ (*x* = 0.8) sodium vanadium fluorophosphate using in situ synchrotron X-ray powder diffraction. J. Mater. Chem. A 2, 7766–7779. 10.1039/C4TA00773E

[B24] Shakoor R. A. Seo D. H. Kim H. Park Y. U. Kim J. Kim S. W. (2012). A combined first principles and experimental study on Na_3_V_2_(PO_4_)_2_F_3_ for rechargeable Na batteries. J. Mater. Chem. 22, 20535–20541. 10.1039/c2jm33862a

[B25] Shen C. Long H. Wang G. Lu W. Shao L. Xie K. (2018). Na_3_V_2_(PO_4_)_2_F_3_@C dispersed within carbon nanotube frameworks as a high tap density cathode for high-performance sodium-ion batteries. J. Mater. Chem. A 6, 6007–6014. 10.1039/C8TA00990B

[B26] Song W. Ji X. Chen J. Wu Z. Zhu Y. Ye K.. (2015). Mechanistic investigation of ion migration in Na_3_V_2_(PO_4_)_2_F_3_ hybrid-ion batteries. Phys. Chem. Chem. Phys. 17, 159–165. 10.1039/C4CP04649H 25372713

[B27] Song W. Ji X. Wu Z. Zhu Y. Li F. Yao Y. (2014). Multifunctional dual Na_3_V_2_(PO_4_)_2_F_3_ cathode for both lithium-ion and sodium-ion batteries. RSC Adv. 4, 11375–11383. 10.1039/C3RA47878E

[B28] Vassilaras P. Ma X. Li X. Geder G. (2013). Electrochemical properties of monoclinic NaNiO_2_. J. Electrochem. Soc. 160, A207–A211. 10.1149/2.023302jes

[B29] Wang L. Lu Y. Liu J. Xu M. Cheng J. Zhang D. (2013). A superior low-cost cathode for a Na-ion battery. Angew. Chem. 125, 2018–2021. 10.1002/ange.201206854 23319239

[B30] Wu L. Hu Y. Zhang X. Liu J. Zhu X. Zhong S. (2018b). Synthesis of carbon-coated Na_2_MnPO_4_F hollow spheres as a potential cathode material for Na-ion batteries. J. Power Sources 374, 40–47. 10.1016/j.jpowsour.2017.11.029

[B31] Wu L. Shi S. Zhang X. Yang Y. Liu J. Tang S. (2018a). Room-temperature pre-reduction of spinning solution for the synthesis of Na_3_V_2_(PO_4_)_3_/C nanofibers as high-performance cathode materials for Na-ion batteries. Electrochim. Acta 274, 233–241. 10.1016/j.electacta.2018.04.122

[B32] Xie Y. Wang H. Liu R. Wang Z. Wen W. Jiang Z. (2017). In situ monitoring of structural and valence evolution during electrochemical desodiation/sodiation process of Na_2_Fe_0.5_Mn_0.5_PO_4_F. J. Electrochem. Soc. 164, A3487–A3492. 10.1149/2.0281714jes

[B33] Xu M. Xiao P. Stauffer S. Song J. Henkelman G. Goodenough J. B. (2014). Theoretical and experimental study of vanadium-based fluorophosphate cathodes for rechargeable batteries. Chem. Mater. 26, 3089–3097. 10.1021/cm500106w

[B34] Yue Y. Binder A. J. Guo B. Zhang Z. Qiao Z. A. Tian C.. (2014). Mesoporous prussian blue analogues: template-free synthesis and sodium-ion battery applications. Angew. Chem. Int. Ed. 53, 3134–3137. 10.1002/anie.201310679 24677672

[B35] Zhao J. Mu L. Qi Y. Hu Y. S. Liu H. Dai S. (2015). A phase-transfer assisted solvo-thermal strategy for low-temperature synthesis of Na_3_(VO_1−x_PO_4_)_2_F_1+2x_ cathodes for sodium-ion batteries. Chem. Commun. 51, 7160–7163. 10.1039/C5CC01504A 25812049

[B36] Zheng J. Yang B. Wang X. Zhang B. Tong H. Yu W. (2018). Comparative investigation of Na_2_FeP_2_O_7_ sodium insertion material synthesized by using different sodium sources. ACS Sustain. Chem. Eng. 6, 4966–4972. 10.1021/acssuschemeng.7b04516

[B37] Zhu C. Wu C. Chen C. C. Kopold P. Aken P. A. V. Maier J. (2017). A high power–high energy Na_3_V_2_(PO_4_)_2_F_3_ sodium cathode: investigation of transport parameters, rational design and realization. Chem. Mater. 29, 5207–5215. 10.1021/acs.chemmater.7b00927

